# Mrgprb2-dependent Mast Cell Activation Plays a Crucial Role in Acute Colitis

**DOI:** 10.1016/j.jcmgh.2024.101391

**Published:** 2024-08-22

**Authors:** Samuel Van Remoortel, Lana Lambeets, Benedicte De Winter, Xinzhong Dong, Juan Pablo Rodriguez Ruiz, Samir Kumar-Singh, Sales Ibiza Martinez, Jean-Pierre Timmermans

**Affiliations:** 1 Laboratory of Cell Biology and Histology and Member of the μNEURO Centre of Excellence, University of Antwerp, Wilrijk, Belgium; 2 Laboratory of Experimental Medicine and Pediatrics and Member of the Infla-Med Centre of Excellence, University of Antwerp, Wilrijk, Belgium; 3 The Solomon H. Snyder Department of Neuroscience, Johns Hopkins University School of Medicine, Baltimore, Maryland; 4 Laboratory of Medical Microbiology, University of Antwerp and Member of the VAXINFECTIO Centre of Excellence, Wilrijk, Belgium

**Keywords:** Colitis, IgE-independent, Mas-related G Protein Coupled Receptor, Mast Cell

## Abstract

**Background & Aims:**

Mast cells (MCs) are typically found at mucosal surfaces, where their immunoglobulin E (IgE)-dependent activation plays a central role in allergic diseases. Over the past years, signaling through Mas-related G protein-coupled receptor b2 (Mrgprb2) in mice and MRGPRX2 in humans has gained a lot of interest as an alternative MC activation pathway with high therapeutic potential. The aim of this study was to explore the relevance of such IgE-independent, Mrgprb2-mediated signaling in colonic MCs in the healthy and acutely inflamed mouse colon.

**Methods:**

Mrgprb2 expression and functionality was studied using a genetic labeling strategy combined with advanced microscopic imaging. Furthermore, Mrgprb2 knockout (Mrgprb2^-/-^) mice were used to determine the role of this pathway in a preclinical dextran sodium sulphate (DSS) colitis model.

**Results:**

We found that Mrgprb2 acts as a novel MC degranulation pathway in a large subset of connective tissue MCs in the mouse distal colon. Acute DSS colitis induced a pronounced increase of Mrgprb2-expressing MCs, which were found in close association with Substance P-positive nerve fibers. Loss of Mrgprb2-mediated signaling impaired DSS-induced neutrophil influx and significantly impacted on acute colitis progression.

**Conclusions:**

Our findings uncover a novel, IgE-independent MC degranulation pathway in the mouse colon that plays a central role in acute colitis pathophysiology, mainly by safeguarding acute colitis progression and severity in mice. This pseudo allergic, Mrgprb2-induced signaling is part of a hitherto unconsidered colonic neuro-immune pathway and might have significant potential for the further development of effective therapeutic treatment strategies for gastrointestinal disorders, such as ulcerative colitis.

## Graphical abstract

**Figure:**
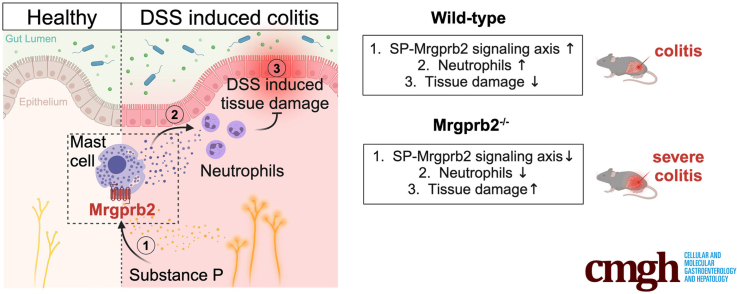

**Summary**

The current study shows the relevance of Mas-related G protein-coupled receptor b2 (Mrgprb2)-dependent, pseudo-allergic mast cell activation in the mouse colon and positions it in a novel SP-Mrgprb2 neuro-immune axis. Moreover, Mrgprb2 signaling acts as an integral part of the dextran sodium sulphate-induced colonic mucosal damage response.

As part of the innate immune system, mast cells (MCs) are typically enriched at mucosal surfaces, including the skin, the airways and the gastrointestinal (GI) tract, where they are ideally positioned to respond to foreign threats.^1^^,^^2^ Canonically, MC activation is understood to result from antigen- immunoglobulin E (IgE) complexes binding to FceRI receptors, which largely depends on the adaptive immune system to provide antigen-specific IgE molecules.^1^^,^^3^ Such IgE-dependent activation leads to massive anaphylactoid degranulation that profoundly impacts tissue functioning and is the central driver of allergic diseases, hence it is often referred to as allergic MC degranulation.^2^

Over recent years, the concept of pseudo-allergic MC activation has gained a lot of interest as an alternative, IgE-independent MC activation paradigm.^4^ Such pseudo-allergic activation does not require prior IgE sensitization but runs through members of the Mas-related G protein-coupled receptor (Mrgpr) family (ie, Mrgprb2 in mice and MRGPRX2 in humans).^5^^,^^6^ Mrgprb2/MRGPRX2 are selectively expressed on the membrane of connective tissue mast cells (CTMCs) and their activation initiates a fast, G protein-mediated signaling cascade that ultimately leads to MC degranulation, which is spatiotemporally distinct from the classical IgE-mediated degranulation.^7^^,^^8^ Characteristically, these receptors are activated by a variety of ligands with cationic properties that are endogenous and exogenous molecules, ranging from (neuro)peptides, such as Substance P (SP), to many United States Food and Drug Administration-approved drugs such as opioids and antibiotics.^9^^,^^10^

Through Mrgprb2/MRGPRX2, MCs are equipped with an effective signaling mechanism that allows them to act as first-line responders to possible threats or damage. Not surprisingly, pseudo-allergic MC activation has redefined our view on MC functioning and currently represents a major target for further therapeutic exploitation.^11^^,^^12^ Studies in the skin have shown that this signaling pathway mediates neurogenic innate immune responses to wounding, protective type 2 inflammation, and antibacterial immunity, but also to pain non-histaminergic itch and even to drug-induced hypersensitivity reactions.^5^^,^^13^, ^14^, ^15^, ^16^ Such broad involvement confirms an essential role in maintaining mucosal homeostasis in the skin, but also raises the question whether Mrgprb2/MRGPRX2 signaling might act as a more widespread mediator that is conserved to other organ systems, yet this remains largely unknown. In this study, we hypothesized that pseudo-allergic signaling is not limited to the skin, but likely plays a relevant role in the colon as well. Therefore, we have characterized the functional role of Mrgprb2 in the mouse colon and studied the importance of this signaling pathway under pathological conditions using a preclinical colitis mouse model.

## Results

### A Novel SP-Mrgprb2 Signaling Axis in the Mouse Distal Colon

**Figure 1:**
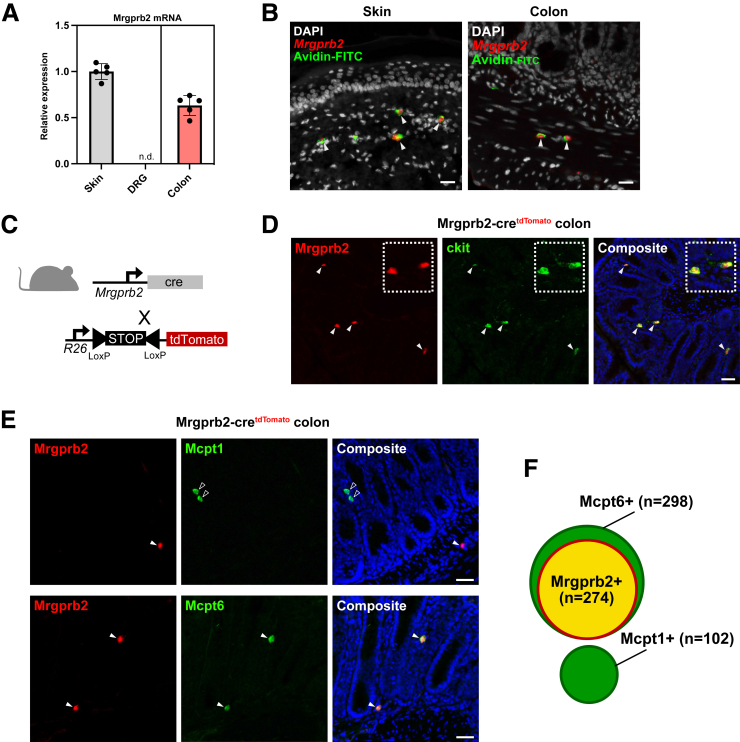
**Mrgprb2 expression in the mouse colon.***A*, Bar chart showing Mrgprb2 mRNA expression as quantified by qPCR in the skin (*left gray bar*), in dorsal root ganglia (*right gray bar*), and colon (*red bar*). Gene expression is normalized to the reference genes *hprt1* and *rps29* and relative to the control group (skin). n.d = not detected. *B*, Representative images of RNAscope in situ hybridization for Mrgprb2 mRNA expression on the skin (*left*) and colon (*right*) of healthy mice. Avidin-FITC (*green*) counterstaining was used as a marker for CTMCs. *C*, Schematic representation of the transgenic Mrgprb2-cre^tdTomato^ mouse design. *D*, Representative image showing Mrgprb2 co-expression in CD117+ colonic MCs (*white arrowheads*). *E*, Mrgprb2+ MCs (*white arrowheads*) do not co-express Mcpt1, a common mucosal MC marker (*open arrowheads*). Mrgprb2+ MCs co-express Mcpt6, a connective tissue MC marker (*white arrowheads*). *F*, Schematic Venn diagram indicating that Mrgprb2 is selectively expressed in the subset of colonic CTMCs. Cell numbers are pooled of 3 animals. Scale bars represent 50 μm.

Mrgprb2 signaling has emerged as a crucial IgE-independent MC activation pathway at mucosal surfaces, yet its expression in colonic MCs remains poorly characterized. Using quantitative PCR (qPCR), we found that, apart from the expected high expression in the skin^5^, Mrgprb2 mRNA is also prominently present in the colon, whereas it was absent in dorsal root ganglia known to lack Mrgprb2 expression^13^ (Figure 1*A*). This was further confirmed using *in situ* hybridization, which equally showed clear Mrgprb2 mRNA expression in both skin and colon (Figure 1*B*). To assess this colonic Mrgprb2 expression in more detail, we bred Mrgprb2-cre^tdTomato^ reporter mice, as these were previously shown to effectively label Mrgprb2+ MCs in the skin^5^ (Figure 1*C*). Using these reporter mice, we found that Mrgprb2+ MCs were also present in the mucosal and submucosal layers of the colon (Figure 1*D*). The colon typically contains 2 major MC subtypes, mucosal MCs (MMCs) and CTMCs, which can be distinguished based on their expression of Mcpt1 and Mcpt6, respectively.^17^ Using these markers, we further characterized which of these MC subsets express Mrgprb2 and found that Mrgprb2 is not expressed in Mcpt1+ MCs (Figure 1*E and F*), but that its expression is highly restricted to Mcpt6-expressing MCs (Figure 1*E and G*), indicating that Mrgprb2 is selectively expressed by colonic CTMCs.

**Figure 2:**
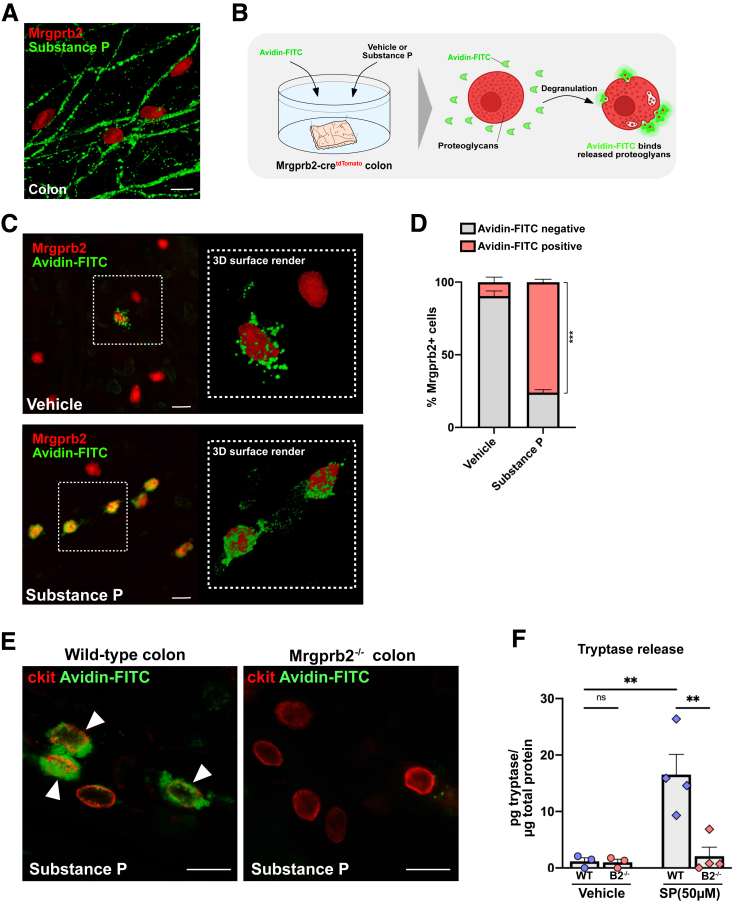
**Characterization of SP-Mrgprb2 signaling axis in the colon.***A*, 3D-rendered image of a (sub)mucosal preparation of Mrgprb2-cre^tdTomato^ colon showing that Mrgprb2-expressing MCs (*red*) are closely associated with SP-containing nerve fibers (*green*). *B*, Schematic representation of the *ex vivo* degranulation imaging principle. Assessment of MC degranulation is frequently used in skin research and is based on the high affinity of fluorescently conjugated Avidin for proteoglycans present in the granules of CTMCs. Upon their degranulation, these proteoglycans are released in the extracellular space and are bound by Avidin-FITC, resulting in the local concentration of Avidin-FITC fluorescence near the cell membrane of MCs *C*, Representative images showing Avidin-FITC labeling of Mrgprb2-expressing MCs in response to vehicle exposure (*upper panel*) or SP (50 μM) exposure (*lower panel*). Scale bars represent 50 μm. *D*, Bar chart showing the percentage of non-degranulating, Avidin-FITC-negative MCs (*gray*) vs degranulating, Avidin-FITC-positive MCs (*red*) upon vehicle or SP (50 μM) exposure. n = 3 animals per treatment. *E*, Representative images showing Avidin-FITC labeling in the colon of Mrgprb2^-/-^ mice. Colonic MCs (CD117+, *red*) in response to SP (50 μM) exposure in the colon of WT mice (*left panel*) and Mrgprb2^-/-^ mice (*right panel*). Colonic MCs of WT mice showed Avidin-FITC labeling, which was abolished in Mrgprb2^-/-^ mice. *F*, Bar chart depicting the amount of tryptase (Mcpt6) release in the medium of WT or Mrgprb2^-/-^ colonic explant cultures during a 1-hour exposure to vehicle or SP (50 μM). Tryptase was markedly increased after SP exposure of WT colon, which was abolished in Mrgprb2^-/-^ colon. Statistical analysis was performed using 1-way ANOVA with Tukey’s post-hoc test (ns: not significant; ∗∗*P* < .01). Scale bars represent 25 μm.

The tachykinergic neuropeptide SP represents an important Mrgprb2 ligand. In the colon, both the intrinsic and extrinsic innervation represent a major source of SP, which led us to further characterize the topological interaction between Mrgprb2+ MCs and SP *in situ*. Using 3-dimensional (3D) tissue imaging, we found that Mrgprb2+ MCs reside in close association to SP+ nerve fibers in the colon (Figure 2*A*). Then, to demonstrate the physiological relevance of SP-Mrgprb2 signaling, we performed degranulation imaging of Mrgprb2+ colonic MCs upon acute SP exposure. To this end, we incubated *ex vivo* (sub)mucosal preparations of Mrgprb2-cre^tdTomato^ colorectum in the presence of Avidin- fluoresceinisothiocyanate (FITC) and exposed them to vehicle or SP (Figure 2*B*). Upon vehicle exposure, the vast majority of Mrgprb2+ MCs were not degranulating (90% ± 4% Avidin-FITC negative) (Figure 2*C and D*). However, SP exposure caused extensive extracellular Avidin-FITC labeling on Mrgprb2+ MCs and robustly increased the proportion of degranulating cells (76% ± 2% Avidin-FITC positive) (Figure 2*C and D*). We verified the specificity of SP-induced degranulation of colonic MCs in Mrgprb2^-/-^ mice and found that SP-mediated degranulation is lost in these mice (Figure 2*E*). Moreover, the amount of tryptase (Mcpt6) released in the medium of colonic explant cultures during a 1-hour exposure to SP (50 μM) was markedly increased in the wild-type (WT) colon, whereas this release was abolished in Mrgprb2^-/-^ colon (Figure 2*F*). Together, these results show that SP-Mrgprb2 represents a functional signaling axis in the colon.

### Acute DSS Colitis Impacts on the SP-Mrgprb2 Axis

**Figure 3:**
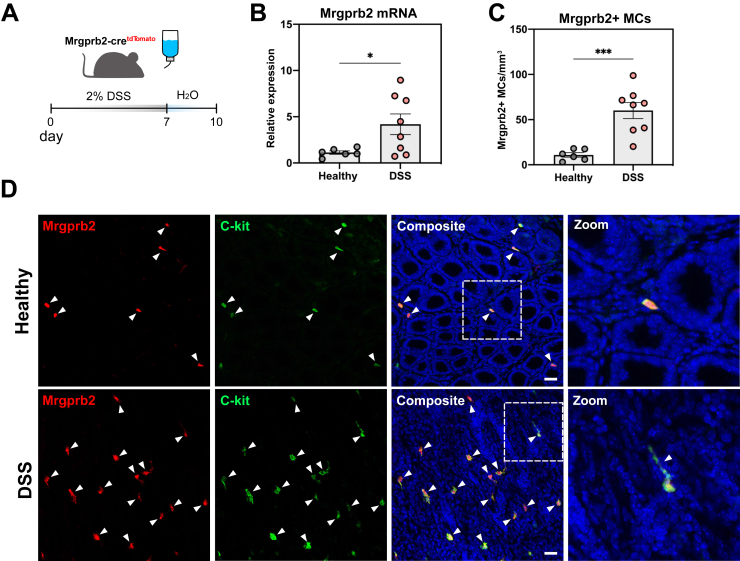
**Acute DSS-induced colitis impacts on Mrgprb2 expression in the colon.***A*, Schematic representation of the DSS treatment regimen in Mrgprb2-cre^tdTomato^ mice. *B*, Bar chart showing Mrgprb2 mRNA expression in the colon of healthy (n = 6) mice and DSS-treated (n = 8) mice. *C*, Bar chart showing the number of Mrgprb2-expressing MCs quantified in submucosal whole mounts of healthy (n = 6) mice and DSS-treated (n = 8) mice. *D*, Representative images of Mrgprb2-expressing MCs in whole mounts of healthy, water-treated mice (*upper panel*) and DSS-treated mice (*lower panels*). Scale bars represent 50 μm. Statistical analysis was performed using a Student’s *t*-test (∗*P* < .05;∗∗∗*P* < .001).

**Figure 4:**
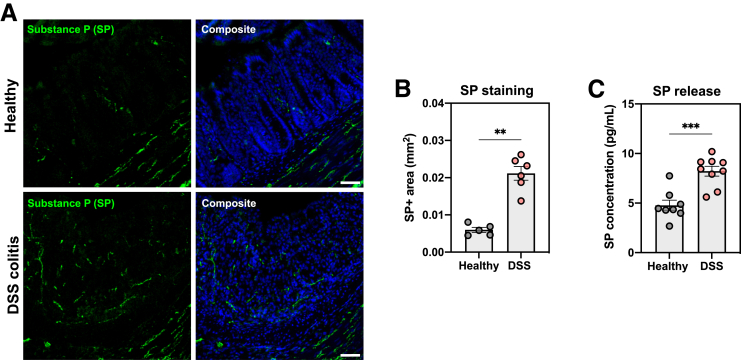
**Acute DSS-induced colitis upregulates SP expression and release in the colon.***A*, Representative images showing SP-immunoreactivity in the colon of healthy (*upper panels*) and DSS colitis mice (*lower panels*). *B*, Bar chart showing SP-immunoreactive area quantified in the colon of healthy WT (n = 5) and DSS-treated WT (n = 6) mice. *C*, Bar chart depicting the amount of SP that was spontaneously released in colonic explant cultures of healthy WT (n = 8) and DSS-treated WT (n = 9) mice. Statistical analysis was performed using a Student’s t-test (∗∗*P* < .01;∗∗∗*P* < .001). Scale bars represent 100 μm.

Having demonstrated the physiological relevance of Mrgprb2-mediated signaling in colonic MCs, we next asked whether this signaling pathway would be affected under pathological conditions. To this end, the well-established dextran sodium sulphate (DSS) colitis model was used in our Mrgprb2-cre^tdTomato^ mice (Figure 3*A*). Upon DSS treatment, we found a significant upregulation of Mrgprb2 mRNA in the colon (Figure 3*B*) and furthermore, a significant increase in the number of Mrgprb2+ MCs (Figure 3*C*). Interestingly, Mrgprb2+ MCs showed a round morphology in the healthy colon, whereas they showed a more ramified morphology upon DSS colitis (Figure 3*D*). Moreover, also SP expression was markedly impacted by acute DSS colitis. Quantification of the SP immunoreactivity area in the colon showed that DSS colitis induced a marked sprouting of SP+ nerve fibers (Figure 4*A and B*). Colonic explant cultures showed that this sprouting was accompanied by a significantly increased SP release in DSS colitis colon compared with healthy colon (Figure 4*C*). Together, these findings show that the SP-Mrgprb2 signaling axis is strongly affected upon acute DSS colitis.

### Loss of Mrgprb2 Signaling Impacts the Progression of DSS Colitis

**Figure 5:**
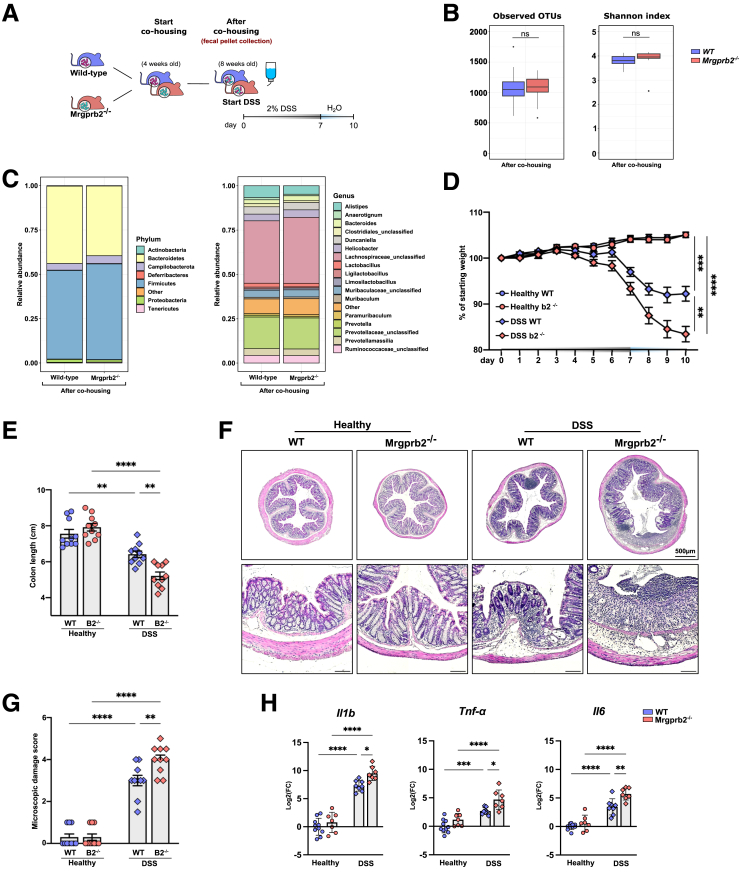
**Acute DSS colitis progression in Mrgprb2**^**-/-**^**mice.***A*, Schematic overview of the experimental setup for co-housing and fecal sampling of WT and Mrgprb2^-/-^ mice before the start of DSS treatment at 8 weeks of age. *B*, Total number of the observed OTUs and Shannon diversity indices for each group (n = 10 mice per group). Data are represented as boxplots. Statistical analysis was performed using pairwise Wilcox testing with Bonferroni *P*-value adjustment (∗∗∗*P* < .001; ns: non-significant). *C,* Group profiles of the relative abundances of microbial species at phylum level (*left*) and genus level (*right*) between WT and Mrgprb2^-/-^ mice before and after co-housing. *D*, Line graph showing body weight changes upon acute DSS colitis development for healthy WT mice (n = 10), healthy Mrgprb2^-/-^ mice (n = 10), DSS-treated WT mice (n = 10), and DSS-treated Mrgprb2^-/-^ mice (n = 10). *E*, Bar chart showing colon length for healthy WT mice (n = 10), healthy Mrgprb2^-/-^ mice (n = 10), DSS-treated WT mice (n = 10), and DSS-treated Mrgprb2^-/-^ mice (n = 10). Statistical analysis was performed using one-way ANOVA with Tukey’s post-hoc test (∗∗*P* < .01; ∗∗∗∗*P* < .0001). *F*, Representative images showing H&E staining on cross sections of the colon of healthy WT mice, healthy Mrgprb2^-/-^ mice, DSS-treated WT mice, and DSS-treated Mrgprb2^-/-^ mice. Scale bars represent 100 μm unless mentioned otherwise. *G*, Bar chart depicting the damage score as quantified by blinded histological assessment in the colon of healthy WT mice (n = 10), healthy Mrgprb2^-/-^ mice (n = 10), DSS-treated WT mice (n = 10), and DSS-treated Mrgprb2^-/-^ mice (n = 10). *H*, Bar charts representing mRNA expression for the pro-inflammatory cytokines il1β, *Tnf-α,* and *Il6* as quantified by qPCR on the colon of healthy WT mice (n = 10), healthy Mrgprb2^-/-^ mice (n = 7), DSS-treated WT mice (n = 8), and DSS-treated Mrgprb2^-/-^ mice (n = 7). Gene expression is normalized to the reference genes *hprt1* and *rps29*. Data are plotted as log 2-fold expression relative to the average of the healthy WT group. Statistical analysis was performed using a Student’s t-test (∗∗*P* < .01) and 1-way ANOVA with Tukey’s post-hoc test (∗*P* < .05; ∗∗*P* < .01; ∗∗∗*P* < .001; ∗∗∗∗*P* < .0001).

Because Mrgprb2-mediated signaling was markedly impacted during colitis, we next sought to interrogate its direct role in the pathophysiology of acute DSS-induced colitis. To address this question, we compared colitis progression between DSS-treated WT and Mrgprb2 knockout (Mrgprb2^-/-^) mice. Prior to the start of the DSS treatment, WT and Mrgprb2^-/-^ mice were co-housed for 4 weeks to equalize the gut microbiome and rule out any pre-existing differences in gut microbiota that could confound our DSS experiments (Figure 5*A*). 16S rRNA sequencing on fecal samples of WT and Mrgprb2^-/-^ mice collected just prior to the start of the DSS experiment showed no significant differences in the total abundance of operational taxonomic units (OTUs) detected in the fecal samples between WT and Mrgprb2^-/-^ mice (Figure 5*B, left panel*). Similarly, the Shannon Diversity Index, which considers both species richness and evenness of the observed OTUs,^18^ revealed no significant differences at the start of the DSS experiment (Figure 5*B, right panel*). Likewise, the distribution of microbial species at both the phylum and genus levels were similar, with similar relative abundances observed between WT and Mrgprb2^-/-^ mice at the start of the DSS treatment (Figure 5*C*).

Under healthy conditions, the Mrgprb2^-/-^ mice did not display any clinical deficits compared with their WT counterparts. However, upon acute DSS-induced colitis, Mrgprb2^-/-^ mice showed significantly more pronounced weight loss (Figure 5*D*). Postmortem analysis showed that DSS-treated Mrgprb2^-/-^ mice had significantly shorter colon lengths (Figure 5*E*) and significantly increased microscopic damage scores (Figure 5*G*). Typically, the colon of DSS-treated Mrgprb2^-/-^ mice showed more extensive mucosal damage, characterized by pronounced ulcerations, loss of epithelial crypts and edema (Figure 5*F*). Moreover, the expression of the hallmark DSS-induced pro-inflammatory cytokines, *Il1β*, *Tnf-α,* and *Il6*, was significantly upregulated in the colon of Mrgprb2^-/-^ mice compared with WT mice, indicating an increased pro-inflammatory response in Mrgprb2^-/-^ mice (Figure 5*H*).

### Loss of Mrgprb2 Signaling Impacts Neutrophil Influx

**Figure 6:**
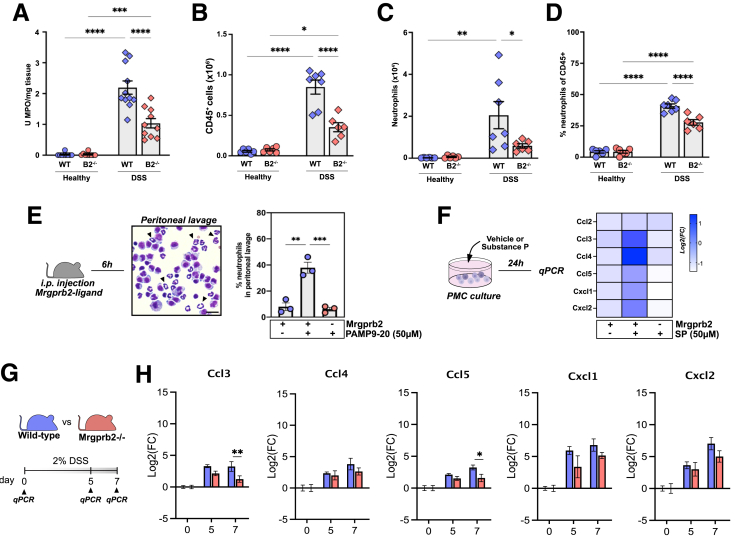
**Loss of Mrgprb2 expression attenuates neutrophil recruitment during DSS colitis progression.***A*, Bar chart showing MPO activity per gram of tissue in the colon of healthy WT mice (n = 9), healthy Mrgprb2^-/-^ mice (n = 9), DSS-treated WT mice (n = 10), and DSS-treated Mrgprb2^-/-^ mice (n = 10). *B*, Bar chart showing the total number of C45+ cells gated from living, single cells in the colon of healthy WT mice (n = 6), healthy Mrgprb2^-/-^ mice (n = 6), DSS-treated WT mice (n = 7), and DSS-treated Mrgprb2^-/-^ mice (n = 6). *C,* Bar chart showing the total number of C45+CD11b+Ly6G+ neutrophils in the colon of healthy WT mice (n = 6), healthy Mrgprb2^-/-^ mice (n = 6), DSS-treated WT mice (n = 7), and DSS-treated Mrgprb2^-/-^ mice (n = 6). *D*, Bar chart showing the proportion of C45+Cd11b+Ly6G+ neutrophils over total CD45+Cd11b+ cells in the colon of healthy WT mice (n = 6), healthy Mrgprb2^-/-^ mice (n = 6), DSS-treated WT mice (n = 7), and DSS-treated Mrgprb2^-/-^ mice (n = 6). *E,* Bar chart showing the percentage of neutrophils in peritoneal lavages 6 hours after intraperitoneal (i.p.) injection of vehicle or PAMP9-20 (synthetic Mrgprb2 ligand, 100 μM). Neutrophils were identified by their typical polymorphonuclear morphology (*black arrows*) after a Diff-Quick stain. PAMP9-20 induced a robust increase in the percentage of neutrophils in the peritoneal lavage in WT mice (*blue circles*), which was abolished in Mrgprb2 knockout mice (*red circles*). *F*, Heat map showing the upregulation of neutrophil-attracting cyto- and chemokine genes in in vitro connective-tissue MC cultures to SP (50 μM) as compared with vehicle-treated cultures (n = 4 independent cultures). Gene expression is normalized to the reference genes *hprt1* and *rps29* and relative to the vehicle-treated group. *G*, Time schedule DSS treatment and timepoints of qPCR analysis performed on tissues isolated on d0, d5, d7 (n = 7–10 mice per timepoint) *H*, The log2-fold change (FC) mRNA gene expression was calculated by normalizing each sample to the average expression level detected in control samples (d0) for Cxcl1, Cxcl2, Ccl3, Ccl4, and Ccl5. Statistical analysis was performed using 2-way ANOVA with Sidak’s post-hoc test. (∗*P* < .05; ∗∗*P* < .01; ∗∗∗*P* < .001; ∗∗∗∗*P* < .0001).

As neutrophil influx is a hallmark of acute DSS colitis pathophysiology,^19^ we determined myeloperoxidase (MPO) activity in colonic homogenates as a readout for neutrophil activity. Interestingly, MPO activity was significantly reduced in DSS-treated Mrgprb2^-/-^ mice as compared with WT mice (Figure 6*A*). In parallel to the reduced MPO activity, we performed flow cytometry to assess neutrophil influx. Overall, CD45^+^ cell counts were significantly reduced in Mrgprb2^-/-^ mice compared with WT mice (Figure 6*B*). When gating for the CD45^+^CD11b^+^Ly6G^+^ neutrophil population, we confirmed that the total number and proportion were significantly reduced in DSS-treated Mrgprb2^-/-^ mice (Figure 6*C and D*). Hence, these findings indicate that Mrgprb2-mediated signaling plays an important role in DSS-induced neutrophil influx.

To further confirm the role of Mrgprb2 signaling in MCs as a driver of neutrophil recruitment, we investigated whether Mrgprb2-mediated MC activation can directly induce neutrophil influx *in vivo*. We treated WT mice intraperitoneally with vehicle or Mrgrpb2 ligand and collected the peritoneal lavage 6 hours later. Assessment of the lavages revealed substantial recruitment of neutrophils into the peritoneal cavity following administration of a Mrgprb2 ligand, but not in vehicle-treated mice (Figure 6*E*). Of note, neutrophil influx upon ligand injection in Mrgprb2^-/-^ mice was minimal, confirming that it is Mrgprb2-mediated (Figure 6*E*). Apart from pre-formed mediators, activated MCs can also *de novo* synthesize innate cytokines and chemokines.^20^ Indeed, treatment of peritoneal MC (PMC) cultures with SP (50 μM) to induce Mrgprb2-mediated activation markedly upregulated the expression of several pro-inflammatory chemokines, including the neutrophil-recruiting cytokines Cxcl1 and Cxcl2 (Figure 6*F*). This effect was Mrgprb2-specific, as PMC cultures derived from Mrgprb2^-/-^ mice did not show such upregulation upon SP stimulation (Figure 6*F*). Hence, these findings confirm that Mrgprb2-mediated signaling in MCs can trigger production of neutrophil-attracting cytokines and induce neutrophil recruitment *in vivo*.

To further explore whether the loss of Mrgprb2 signaling in MCs also impaired the production of neutrophil-attracting cytokines upon DSS-induced damage, we determined their expression during the active DSS exposure phase where epithelial damage is most active (ie, 5 days and 7 days) (Figure 6*G*). Interestingly, whereas the colon of DSS-treated WT mice showed upregulation of *Ccl3, Ccl4, Ccl5, Cxcl1, and Cxcl2* at days 5 and 7, these expressional changes were markedly attenuated in colon of DSS-treated Mrgprb2^-/-^ mice, further supporting an impaired neutrophil recruitment (Figure 6*H*). Thus, Mrgprb2-mediated MC activation drives neutrophil recruitment through cytokine/chemokine production and, especially during the early phase of DSS-induced damage, loss of Mrgprb2 signaling influences neutrophil recruitment to the DSS-induced colonic damage.

### Enhanced Pro-inflammatory Response Due to Loss of Mrgprb2 Signaling is Associated With Macrophage Activation and Tissue Remodeling

**Figure 7:**
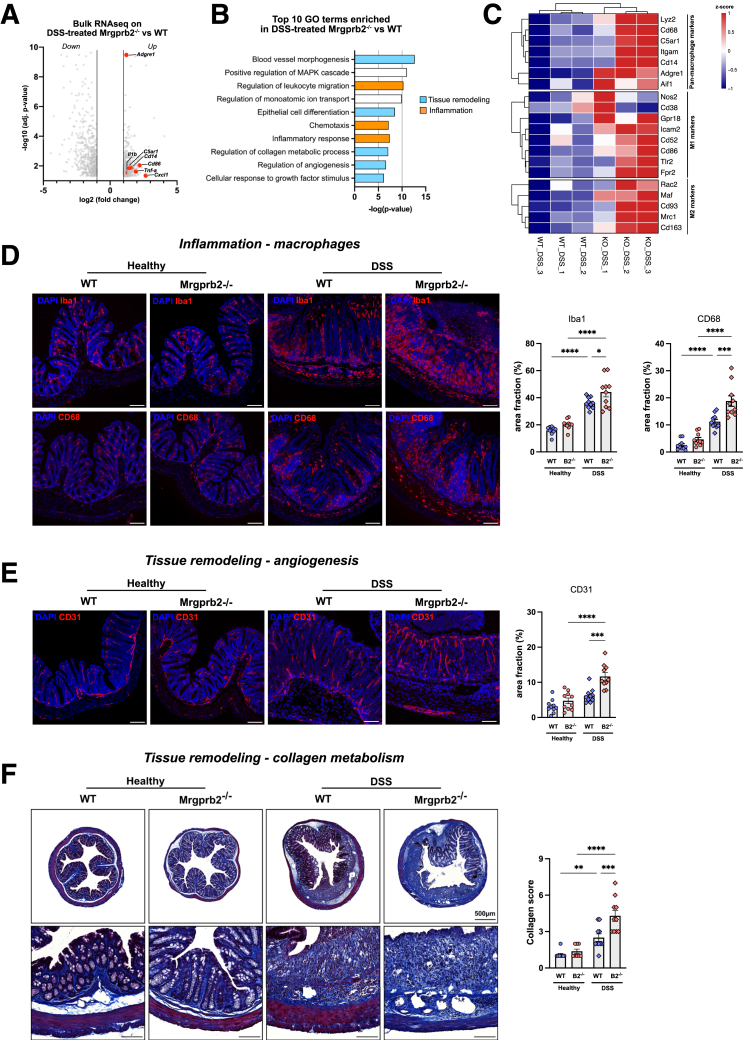
**Characterization of the underlying drivers of worsened colitis progression in Mrgprb2**^**-/-**^**mice.***A*, Volcano plot showing differentially expressed genes (DEGs) with a log2 fold change >1 in the colon of DSS-treated Mrgprb2^-/-^ mice compared with WT mice. Highlighted genes (*red dots*) pertain to macrophages and their activation. *B*, Bar chart showing the top 10 gene ontology terms enriched in the transcriptomic signature of DSS-treated Mrgprb2^-/-^ mice as compared with DSS-treated WT mice. Terms highlighted in *light blue* are related to tissue remodeling, terms highlighted in *orange* are related to inflammation. *C*, Clustered heatmap of DEGs related to macrophage functioning from RNA-seq analysis. *D*, Representative images showing Iba1 and CD68 immunoreactivity in the colon of DSS-treated WT mice (*left panel*) and DSS-treated Mrgprb2^-/-^ mice (*right panel*). Bar chart shows the Iba1-immunoreactive area in the colon of DSS-treated WT (n = 10) mice and DSS-treated Mrgprb2^-/-^ (n = 10) mice. *E*, Representative images showing CD31 immunoreactivity in the colon of DSS-treated WT mice (*left panel*) and DSS-treated Mrgprb2^-/-^ mice (*right panel*). Bar chart shows the CD31-immunoreactive area in the colon of DSS-treated WT (n = 10) mice and DSS-treated Mrgprb2^-/-^ (n = 10) mice. *F*, Representative images of Trichrome Masson-stained sections of the colon of DSS-treated WT mice (*left panel*) and DSS-treated Mrgprb2^-/-^ mice (*right panel*). Bar chart shows collagen deposition scoring in the colon of DSS-treated WT (n = 10) mice and DSS-treated Mrgprb2^-/-^ (n = 10) mice. Statistical analysis was performed using a Student’s *t*-test (∗∗*P* < .01) and 1-way ANOVA with Tukey’s post-hoc test (∗*P* < .05; ∗∗*P* < .01; ∗∗∗*P* < .001; ∗∗∗∗*P* < .0001). Scale bars represent 100 μm.

Loss of Mrgprb2 expression strongly aggravates colitis outcome, which is associated with impaired neutrophil recruitment, yet the underlying mechanism underlying this worsened outcome is unclear. To obtain an unbiased view, we performed bulk RNA sequencing on the colon of DSS-treated WT and Mrgprb2^-/-^ mice. In comparison with DSS-treated WT mice, the loss of Mrgprb2 signaling results in profound transcriptional changes upon DSS colitis (Figure 7*A*). Gene ontology (GO) analysis revealed 2 major transcriptional signature changes in Mrgprb2^-/-^ mice, being tissue remodeling (ie, angiogenesis and collagen metabolism) and inflammation (Figure 7*B*).

First, we sought to validate the altered tissue remodeling response in Mrgprb2^-/-^ mice in more detail. PECAM1/CD31 immunohistochemistry revealed markedly increased blood vessel staining in the colon of Mrgprb2^-/-^ mice, especially in the mucosal area of ulcerated regions, confirming that loss of Mrgprb2 expression leads to profound blood vessel remodeling (Figure 7*E*). Moreover, Trichrome-Masson collagen visualization showed strongly increased collagen deposition in mucosal and submucosal regions of Mrgprb2^-/-^ mice, as well as marked distortion of the muscularis layer, further confirming marked tissue remodeling in Mrgprb2^-/-^ mice (Figure 7*F*). Overall, these findings confirm our RNAseq findings at tissue level and expose an important role for Mrgprb2 in controlling acute DSS colitis progression.

With regard to the inflammatory gene signature, we noticed that several genes associated with macrophages and their functioning were markedly altered in DSS-treated Mrgprb2^-/-^ mice, such as the classical pan-macrophage markers *Adgre1 (*ie, F4/80), *C5ar1,* and *Cd14 (*markers associated with macrophage activation pathways), were upregulated in DSS-treated Mrgprb2^-/-^ mice compared with DSS-treated WT mice. Additionally, a selection of markers typically associated with M1 activation (ie, *Gpr18, Icam2, Cd52, Cd86, Tlr2, and Fpr2*) and M2 activation (ie, *Rac2, Maf, Cd93, Mcr1, Cd163)* were upregulated in DSS-treated Mrgprb2^-/-^ mice compared with DSS-treated WT mice (Figure 7*C*). To further confirm the involvement of macrophages, we stained for the pan-macrophage markers Iba-1 and CD68 and found that DSS-treated showed significantly increased Iba-1 and CD68 staining compared with their WT controls (Figure 7*D*). Of note, no differences were seen between healthy WT and healthy Mrgprb2^-/-^ mice (Figure 7*D*).

### Impaired Neutrophil Influx Causes Abnormal Macrophage Influx During DSS Colitis

**Figure 8:**
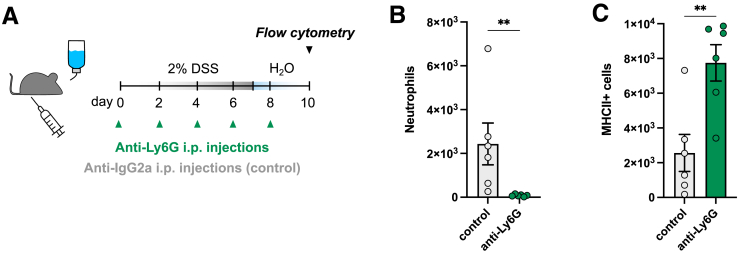
**Neutrophil depletion during DSS colitis causes abnormal macrophage influx.***A,* Time schedule DSS treatment and timepoints of anti-Ly6G injections (every other day until analysis on day 10). Each mouse received a total of 5 injections. *B–C*, The total number of C45^+^Cd11b^+^Ly6G^+^ neutrophils and CD45^+^CD11b^+^Ly6g^-^CD64^+^Ly6c^-^MHCII^+^ macrophages in the colons of anti-Ly6G treated WT mice (n = 6) and anti-IgG2a treated control WT mice (n = 6) analyzed by flow cytometry. Statistical analysis was performed using a Student’s *t*-test (∗∗*P* < .01).

Our results demonstrate that the absence of Mrgprb2-mediated neutrophil recruitment results in immune imbalance and inappropriate inflammatory responses, characterized by an increase release of pro-inflammatory cytokines, aberrant tissue remodeling, and a pronounced increase of macrophage markers. To add further evidence that an impairment of neutrophil influx during active DSS-induced damage can drive the increased macrophages observed in our RNAseq and immunohistochemistry (IHC) data at day 10, we performed anti-Ly6G antibody neutrophil depletion experiments during the DSS protocol and analyzed the resulting effect on macrophages via flow cytometry (Figure 8*A*). Anti-Ly6G treatment effectively resulted in the depletion of neutrophils in the DSS affected colons (Figure 8*B*). Interestingly, neutrophil depletion during DSS-induced colitis caused a significant increase in number of macrophages in the colon, as evidenced by the increased MHCII+ macrophage population in the anti-Ly6G-treated mice as compared with vehicle-treated mice (Figure 8*C*). Mechanistically, this further strengthens our hypothesis that impaired neutrophil influx during active DSS-induced damage can drive the abnormal increases in macrophages as observed in Mrgprb2^-/-^ mice.

## Discussion

The pseudo-allergic, Mrgprb2/MRGPRX2-mediated signaling pathway has emerged as a novel, IgE-independent MC degranulation pathway being of great importance in the skin. In the present study, we have shown that this signaling pathway is highly relevant in colonic MCs as well. Using a preclinical acute colitis model, we provided clear evidence that this signaling pathway plays an essential role in acute DSS-induced colitis.

We demonstrated that Mrgprb2 is selectively expressed in a subset of colonic MCs, namely the CTMCs. As opposed to MMCs, which are bone marrow-derived and rather short-lived, recent evidence indicates that CTMCs are embryonically seeded and long-lived tissue resident MCs that start expressing Mrgprb2 already early in life, indicating its role as a constitutive signaling pathway in colonic MCs.^17^^,^^21^ Furthermore, we showed that Mrgprb2-positive MCs closely associate with nerve fibers expressing one of the hallmark ligands of Mrgprb2 (ie, the neuropeptide SP). Moreover, activation of Mrgprb2-expressing MCs by SP *in situ* directly induced their degranulation in an Mrgprb2-dependent manner. Collectively, these findings for the first time show the existence of a functional SP-Mrgprb2 signaling axis in the mouse colon that mediates IgE-independent MC degranulation. Whereas canonical IgE-mediated signaling requires prior immune-mediated IgE-production, such SP-Mrgprb2 signaling allows colonic MCs to directly degranulate upon SP exposure, making it an effective first-line defense mechanism in the colonic neuro-immune environment. This is comparable to the situation in the skin, where Mrgprb2+ MCs form intricate neuro-immune units with SP-expressing sensory neurons that regulate neurogenic inflammation, itch, and pain.^13^^,^^15^^,^^16^

Our study confirms that Mrgprb2 signaling plays a crucial role in acute DSS colitis pathophysiology. On the one hand, we found a significant increase in the number of Mrgprb2+ MCs in the DSS-affected colon, indicating that Mrgprb2-expressing MCs likely take part in acute colitis. On the other hand, Mrgprb2 knockout profoundly impacted on the DSS colitis progression. Whether the increased number of Mrgprb2-expressing MCs is due to local proliferation, as part of their long-lived tissue resident phenotype,^21^ or rather due to an influx of bone marrow-derived precursors remains unclear. Both phenomena were recently reported to drive local MC expansion in an inflammation-induced atopic dermatitis model.^22^ Evidently, our findings raise the question on how increased Mrgprb2 signaling would be triggered during acute colitis, because an increased number of Mrgprb2-positive MCs does not necessarily reflect increased Mrgprb2 signaling. Yet, both the expression and release of its endogenous ligand SP was strongly upregulated upon acute colitis in our model, supporting the hypothesis that SP could be an important mediator that drives increased Mrgprb2 signaling in acute DSS colitis. This is further supported by the well-known role of upregulation of SP in the pathophysiology of colitis,^23^^,^^24^ especially in immune modulation, further supporting our hypothesis that part of the actions of SP could be mediated via Mrgprb2 expressed on MCs, which in turn is important for mounting an appropriate tissue immune response. Interestingly, it was recently described that nociceptor-derived SP exert a protective role in acute colitis, mainly via its effect on the gut microbiota and preventing dysbiosis.^25^ Whether SP could exert its effect on the gut microbiota through Mrgprb2 signaling on MCs remains unclear and launches an exciting topic for future research. Moreover, although SP remains the most probable mediator acting on Mrgprb2, given the close association of Mrgprb2-expressing MCs and SP-expressing nerve fibers, we cannot rule out that other known Mrgprb2 ligands, such as pro-adrenomedullin (PAMP20),^15^ bacterial quorum sensing molecules^14^ and/or anti-microbial peptides^26^ are also present in the colon micro-environment and induce increased Mrgprb2 signaling.

Remarkably, our findings expose a previously unrecognized role for colonic MCs during the DSS-induced mucosal immune response. In addition to the rapid release of pre-formed MC mediators such as histamine and tryptase, our *in vitro* and *in vivo* experiments show that Mrgprb2-mediated activation of MCs leads to the production of several cytokines and chemokines that induce neutrophil recruitment in an Mrgprb2-dependent manner. Moreover, such Mrgprb2-dependent signaling in MCs seems to play a crucial role in neutrophil recruitment in DSS-induced colitis, as evidenced by the attenuated production of neutrophil- recruiting cytokines in the early phases of DSS-induced colitis in Mrgprb2^-/-^ mice, as well as the strongly impaired neutrophil influx in DSS-treated Mrgprb2^-/-^ mice. Upon DSS-induced damage, neutrophils are recruited to the injured colonic mucosa, where they act as first-line responders that serve to combat the infectious threats and thereby prevent excessive tissue damage.^27^^,^^28^ Ultimately, this suggests that Mrgprb2-mediated signaling in colonic MCs acts as an important player for triggering this adequate protective immune response upon DSS-induced tissue damage. This proposed pathway in the gut seems very parallel to the skin, where Mrgprb2-mediated signaling in MCs plays a similar protective role as driver of damage-induced neutrophil influx.^13^^,^^29^

This protective role in the DSS-induced tissue damage response is further evidenced by our findings that overall colitis progression is progressively worsened when Mrgprb2 signaling in colonic MCs is impaired. Of note, although our WT and Mrgprb2^-/-^ mice were not born littermates, we did not observe any differences in fecal microbiota composition after co-housing from weaning until the start of the experiments, ruling out that the observed differences in colitis progression between WT and Mrgprb2^-/-^ mice are confounded by pre-existing differences in gut microbiota. Interestingly, our findings are in line with recent reports describing similar impairments in innate immune responses and worsened DSS-induced tissue damage upon Mrgprb2 expression loss.^30^^,^^31^ However, whereas an impaired immune response was linked to increased oxidative stress and disturbed barrier function specifically in the early phases of DSS-induced damage (ie, during active DSS exposure for 7 days), our findings add further evidence that these early impairments have profound detrimental effects on the later progression and resolution of DSS-induced damage (ie, after 7 days active DSS exposure and 3 days recovery more specifically by setting the stage for an exacerbated pro-inflammatory response), as evidenced by the upregulation of hallmark pro-inflammatory cytokines such as Il-1β, Tnf-α, and Il-6, and worsened clinical outcome. Of note, studies using antibody-mediated neutrophil depletion,^32^, ^33^, ^34^ and genetic depletion strategies^35^^,^^36^ have shown that impaired neutrophil influx similarly worsens colitis outcome, further underlining that impaired neutrophil influx caused by loss of Mrgprb2-mediated signaling in MCs can indeed contribute to the aggravated colitis outcome.

Further molecular characterization of the worsened colitis progression via RNA sequencing revealed that loss of Mrgprb2 signaling strongly changes colitis progression towards a pro-inflammatory and pro-fibrotic tissue response. This was characterized by an exacerbated pro-inflammatory response, abnormal angiogenesis, and collagen deposition, which are all associated with aggravated progression in patients with ulcerative colitis (UC) and are considered important risk factors for chronic intestinal inflammatory disorders and fibrosis.^37^, ^38^, ^39^, ^40^ Interestingly, our findings also reveal a strongly upregulated macrophage functioning in the colon of DSS-treated Mrgprb2^-/-^ mice, as evidenced by an upregulation of genes associated with macrophages and their functioning, as well as an increased presence of macrophages as shown by immunohistochemical staining for classical pan-macrophage markers Iba-1 and CD68. Interestingly, when we further investigated the expression of a selection of markers typically associated with M1 and M2 polarization. We found that several M1 markers (Gpr18, Icam2, Cd52, CD86, Tlr2, and Fpr2) as well as M2 markers (Rac2, Maf, CD93, Mcr1, Cd163) were upregulated in the DSS-treated Mrgprb2^-/-^ mice, suggesting that the upregulated macrophages exhibit a spectrum of activation states rather than fitting neatly into either the M1 or M2 phenotype. This aligns with recent observations that the M1–M2 classification is an oversimplification and that macrophages can display mixed M1/M2 phenotypes, expressing markers characteristic of both subsets, especially in complex environments, like tumor microenvironments.^41^^,^^42^ In this respect, further studies into these macrophage phenotypical changes are warranted, especially into their possible role in creating the exaggerated pro-inflammatory environment. Nevertheless, our findings clearly indicate that loss of Mrgprb2 expression disturbs the innate immune balance in acute DSS colitis, leading to an exaggerated pro-inflammatory response that, at least in part, is driven by abnormally increased macrophage functioning.

We do realize that this study is exclusively focused on the mouse Mrgprb2 receptor and might raise the question about the possible translational potential of our findings to the human gut. In this respect, it is worthwhile to mention that the expression of its human counterpart, MRGPRX2, has also been described in colonic MCs.^21^^,^^43^ Interestingly, our study employs a genetic knockout strategy that results in the lack of functional Mrgprb2 expression, but several single nucleotide polymorphisms (SNPs) have been identified in the human *MRGPRX2* gene that have similar ‘loss of function’ effects on MRGPRX2 functioning.^44^ One of the most common loss of function SNPs in *MRGPRX2* (rs10833049) is a missense mutant that causes replacement of asparagine by serine at position 62 of the protein (N62S) and consequently impairs SP-induced MRGPRX2 activation and MC degranulation.^45^ Strikingly, a recent study found that this SNP is significantly enriched in a Jewish population with 4-fold increased prevalence of UC, linking impaired MRGPRX2 signaling to UC pathophysiology.^46^ These findings in human strongly parallel our observations in mice and support the hypothesis that pseudo-allergic MC activation is an integral part of UC pathophysiology, warranting further translational studies into the possible therapeutic potential of MRGPRX2 signaling in UC.

In conclusion, the current study shows the relevance of Mrgprb2-depdendent, pseudo-allergic MC activation in the mouse colon, and positions it in a novel SP-Mrgprb2 neuro-immune axis. Moreover, Mrgprb2 signaling acts as an integral part of the DSS-induced colonic mucosal damage response, where the receptor is essential for mounting an appropriate colonic damage response to prevent excessive tissue damage and inflammation. From a broader perspective, our study opens novel research avenues for pseudo-allergic MC activation in UC, but also other GI-related conditions with known MC involvement.

## Methods

### Mice

C57BL6J WT mice (originally from Charles River, bred in-house) and C57BL/6J Mrgprb2 knockout mice (Mrgprb2^-/-^, kindly provided by Prof. Xinzhong Dong, bred in house) were both bred and maintained in a conventional animal facility. Mrgprb2-cretdTomato mice were generated by breeding Mrgprb2-cre mice (kindly provided by Prof. Xinzhong Dong) with R26-LSL-tdTomato reporter mice (JAX strain 7909). All animals used were age- and sex-matched for experiments. Genotyping was performed as described previously.^5^ Animal housing and handling procedures were conducted in accordance with the European Directive 86/609/EEC and approved by the Ethical Committee for animal experiments of the University of Antwerp (EC 2020-50)

### DSS Colitis

Because independent lineages of animals can display differences in the composition of gut microbiota, potentially leading to variations in DSS-induced colitis phenotypes,^47^ we co-housed the WT and Mrgprb2^-/-^ mice for 4 weeks before the start of DSS treatment at 8 weeks of age. This strategy has been previously shown to equalize the gut microbiome by sharing microbes across the co-housed experimental mice.^19^ At the start of the experiment, mice received autoclaved drinking water (control) or 2% DSS in autoclaved drinking water (DSS) for 7 days ad libitum, followed by a 3-day wash-out period with normal autoclaved water. Only male mice were used to avoid variability and reproducibility issues known to occur in female mice.^19^ Mice were followed up individually every day for weight loss, stool consistency, and rectal bleeding.

### 16S rRNA Sequencing

Metagenomic DNA was extracted from fecal pellets using the FastDNA Spin Kit for Feces (MP Biomedicals) according to the manufacturer’s instructions. Extracted DNA was quantified with the Qubit dsDNA HS Assay Kit with a Qubit 4.0 Fluorometer (ThermoFisher Scientific). The average DNA concentration of samples was 81.0 ± 60.6 ng/μL. PCR amplification of the V3–V4 regions of the 16S rRNA gene was performed in duplicate for each sample using Illumina fusion primers (341F and 802R) with 2x KAPA HiFi Hot Start Ready Mix (Roche) with initial denaturation at 95 °C for 3 minutes; 25 cycles of 95 °C for 30 seconds, 60 °C for 30 seconds, and 72 °C for 30 seconds; and elongation at 72 °C for 10 minutes. Multiplexed 16S rRNA gene libraries were prepared using the Nextera XT kit (Illumina Inc), followed by 2 × 250 paired-end sequencing performed with V2 chemistry on a MiSeq instrument (Illumina Inc). Reads were trimmed with TrimGalore v.0.6.7 using quality score of 30. Trimmed reads were aligned to the SILVA database v.132 and clustered into OTUs using mothur v1.44.1. Sequencing error rate was assessed with mothur by incorporating a well-characterized mock community (HM-783D, BEI Resources), resulting in an error rate of 0.036%. Negative controls during extraction and library preparation were included and used to remove contaminants from the OTU table with decontam v.1.14.0. Phyloseq v.1.38.0 was used for rarefaction of the number of reads across the samples to an even depth using seed 711. Shannon index was obtained using vegan v.2.6.4. Normality of α-diversity was assessed with the Shapiro-Wilk test, and comparisons between groups were performed using pairwise Wilcox testing with Bonferroni *P*-value adjustment. (∗*P* < .05; ∗∗*P* < .01; ∗∗∗*P* < .001; ∗∗∗∗*P* < .0001).

### Histopathological Scoring

Colon tissue was isolated and processed for paraffin embedding. Full-circumference colon (1 cm) was immersion-fixed in 4% paraformaldehyde (4% PFA, in 0.01M phosphate buffered saline [PBS], pH 7.4) for 2 hours at room temperature (RT) and processed for paraffin embedding. Five-μm paraffin sections of 2 distinct regions were mounted on glass microscopic slides and were stained with hematoxylin and eosin (H&E) or Trichrome Masson for collagen deposition scoring. H&E-stained slides were used for histological assessment of DSS colitis progression using a standardized scoring system.^19^ Briefly, overall tissue damage was scored: no damage (0), isolated focal epithelial damage (1), mucosal erosions and local ulcerations (2), and extensive mucosal damage with large ulcerations (3), as were lamina propria immune cell infiltrates: none (0), increased (1), submucosal presence of local immune cell clusters (2), and extensive presence of transmural immune cell infiltration (3). Scores for tissue damage and immune cell infiltrates were summed, resulting in a cumulative score ranging from 0 to 6. Trichrome Masson-stained slides were used for histological assessment of DSS-induced collagen deposition using a standardized scoring system.^48^ Briefly, the degree of collagen deposition was scored: normal submucosa (0), increased in submucosa (1), increased in mucosa (2), increased in muscularis mucosa, with disorganization of muscularis mucosa (3), increased in muscularis externa (4), and gross deposition and disorganization of muscularis externa (5), as was the percent involvement per section: 1% to 25% (1), 26% to 50% (2), 51% to 75% (3), 76% to 100% (4). Both scores were summed, resulting in a cumulative score ranging from 1 to 9. Histological scoring was performed by an experimenter blinded for groups.

### IHC and Imaging

Colonic tissues were isolated and processed for IHC on frozen sections (IHC-Fr) or on whole mounts (IHC-WM). For IHC-Fr, colon was immersion-fixed in 4% PFA for 2 hours at RT. Subsequently, tissues were incubated overnight in a 30% sucrose solution and embedded in Optimal Cutting Temperature medium. For each tissue, 4 cryosections (12-μm-thick sections, with 250 μm in between sections) were collected on Superfrost slides and dried for 2 hours at 37 °C. Next, tissue sections were permeabilized and blocked for 1 hour with PBS containing 1% Triton X-100 and 10% normal horse serum at RT, followed by an overnight incubation with primary antisera (Table 1) at RT. After washing, secondary antisera (Table 1) were applied to the tissues for 2 hours at RT. All antisera were diluted in 0.01 M PBS containing 10% normal horse serum and 1% bovine serum albumin. High-resolution images were obtained on a Leica TCS SP8 laser scanning confocal microscope. For quantification of staining area, 5 random non-overlapping fields of view were imaged per section, then a threshold was applied, and the fraction of staining area was calculated over the total staining area. For IHC-WM, the colon was opened along the mesenteric border and pinned flat in a Sylgard-coated petri dish, followed by fixation with 4% PFA for 2 hours at RT. Next, (sub)mucosal preparations were obtained by gently removing the tunica muscularis under a binocular microscope and further processed for staining and imaging. Antibody staining was performed using the same protocol as described above (Table 1). To allow optimal in toto imaging, (sub)mucosal preparations were optically cleared using the refraction-index matching CE3D protocol and imaged on a Leica SP8 confocal microscope system using a 20× glycerol-immersion objective.^49^ To quantify the number of Mrgprb2+ MCs, 20 randomly chosen fields of view were imaged, after which the number of cells per imaging volume were determined.

**Table 1:** List of Primary and Secondary Antibodies

Tissue stainings		
Primary antibody	Species/clonality	Vendor/catalog
c-Kit (cd117)	Goat polyclonal	R&D AF1356
Mcpt1 (chymase)	Sheep polyclonal	R&D AF5146
Mcpt6 (tryptase)	Rat monoclonal	R&D MAB3736
Substance P	Guinea pig polyclonal	Abcam ab10353
CD31/PECAM1	Rat monoclonal	Abcam ab56299
Iba-1	Rabbit polyclonal	WAKO 019-19741
CD68	Rat monoclonal	Bio-Rad MCA1957

Secondary antibody	Vendor/catalog	
Donkey-anti-Goat-FITC	Jackson 705-095-003	
Donkey-anti-Sheep-Cy5	Jackson 713-175-147	
Donkey-anti-Rat-FITC	Jackson 712-095-153	
Donkey-anti-Guinea pig-FITC	Jackson 706-095-148	
Donkey-anti-Rat-Cy3	Jackson 712-165-153	
Donkey-anti-Rabbit-Cy3	Jackson 711-165-152	

Flow cytometry stainings		
Antibody (clone)	Conjugate	Vendor/catalog
CD45 (30-F11)	APC-Cy7	eBioscience 47-0451-82
CD11b (M1/70)	PE-Cy7	BioLegend 101215
Ly6G (IA8)	FITC	BioLegend 127625
CD64 (X54-5/7.1)	BV711	BioLegend 139311
Ly6c (HK1.4)	APC	BioLegend 128015
MHCII (M5/114.15.2)	BV421	BioLegend 107631

### RNA Scope

To localize Mrgprb2 mRNA expression, in situ hybridization was performed using the RNAScope 2.5 HD RED platform (Advanced Cell Diagnostics). All tissue preparations and experiments were carried out according to the manufacturer’s protocol. Briefly, isolated tissues were immersion-fixed in 4% paraformaldehyde (4% PFA, in 0.01M PBS, pH 7.4) for 24 hours at RT and processed for paraffin embedding. Five-micron-thick paraffin sections were mounted on Superfrost glass slides (Thermo Fisher Scientific) and tissue sections underwent standard pretreatment, followed by incubation for 2 hours with the target probe. For detection of Mrgprb2 mRNA, probe reactivity was visualized with Fast Red, and sections were counterstained with Avidin-FITC and DAPI to visualize connective-tissue MCs and nuclei, respectively. Images were obtained on a Leica SP8 confocal microscope system.

### Ex Vivo MC Degranulation Assay

Full-circumference colon (3 cm) was placed in ice-cold Krebs buffer oxygenated with 95% O_2_/5% CO_2_, after which it was opened along the mesenteric line and the *tunica muscularis* was gently removed under a stereomicroscope leaving the mucosa/submucosa intact. Mucosa/submucosa preparations were pinned flat in a Sylgard-coated petri dish with the submucosa facing up and incubated for 10 minutes with Avidin-FITC (100 μg/ml, Merck-Millipore) in Krebs buffer at 37 °C. Next, either vehicle or the Mrgprb2 ligand SP (50 μM, Tocris) was added, and the preparations were incubated for 35 minutes at 37 °C. After incubation, preparations were shortly rinsed with Krebs buffer and fixed with 4% PFA solution for 18 to 24 hours at 4 °C. To allow for whole tissue imaging, fixed preparations were optically cleared using the refraction-index matching CE3D protocol^49^ and imaged on a Leica SP8 using a 20×/0.15 and a 63×/1.20 immersion objective for overview and high-resolution images, respectively. Images were recorded from three biological replicates per group, with 20 imaged regions per biological replicate. Mrgprb2+ MCs were identified based on their cytoplasmatic tdTomato signal, and the presence of at least 3 dense Avidin-FITC spots near the MC membrane was used as degranulation threshold. The percentage of degranulating versus non-degranulating tdTomato+ MCs was determined for each preparation, and all images were analyzed blinded. Three-D rendered images were generated using the Leica Application suite (LAS X) software.

### Measurements of Protein Released From Colonic Explant Tissue

For SP release, full-circumference colon (3 cm) was placed in a 12-well plate containing 1.5 ml of RPMI 1640 medium supplemented with 10% fetal bovine serum (FBS), 1% antibiotic/mycotic, and 10 μg/ml gentamycin) and incubated for 6 hours at 37 °C. For Mcpt6 release, full-circumference colon (3 cm) was placed in ice-cold Krebs buffer oxygenated with 95% O_2_/5% CO_2_, after which it was opened along the mesenteric line, and the tunica muscularis was gently removed under a stereomicroscope, leaving the mucosa/submucosa intact. Next, mucosa/submucosa preparations were placed in a 12-well plate containing 1.5 ml Krebs buffer and incubated for 1 hour at 37 °C in the presence of vehicle or the Mrgprb2 ligand, SP (50 μM, GenScript). After incubation, tissue supernatant was collected and centrifuged at 10.000 g for 10 minutes, followed by storage at −80 °C. Mcpt6 and SP protein concentration were determined using the Tpsb2/Mcpt6 ELISA kit (Cusa Bio) and Substance P ELISA kit (Cayman Chemical), respectively, according to the manufacturer’s protocol.

### In vivo Mrgprb2-mediated Peritoneal Neutrophil Recruitment

WT and Mrgprb2^-/-^ mice were injected intraperitoneally with vehicle (saline) or 50 μM PAMP9-20 (custom-made at GenScript). Six hours after the treatment, mice were sacrificed using cervical dislocation, abdominal skin was opened, and 2 sequential lavages were performed by injecting 6 mL HBSS+/+ (supplemented with 3% FBS and 0.01M HEPES) in the peritoneal cavity using a 10 mL syringe equipped with a 27 G needle. Peritoneal cells were subsequently collected in a 15 mL tube and centrifuged for 5 minutes, 300 g at RT. Cells were counted and dissolved at a density of 1 × 106 cells in PBS (1% bovine serum albumin). Cytospins were obtained using a Shandon cytospin centrifuge by centrifuging 100 μl of the cell suspension on Superfrost glass slides (Thermo Fisher Scientific) at 800 RPM for 5 minutes. Air dried cytospins were stained with a Diff-Quik stain (RAL diagnostics). Neutrophils were identified by their typical polymorphonuclear morphology, and the percentage of neutrophils was calculated per sample.

### PMC Cultures

PMCs were obtained as described previously.^50^ Briefly, after peritoneal lavage, the obtained cells were cultured at a density of 1 × 10^6^ cells/ml in RPMI 1640 medium supplemented with 10% FBS, penicillin-streptomycin (100 IU/ml), recombinant mouse IL-3 (10 ng/ml), and SCF (30 ng/ ml) (37 °C and 5% CO_2_). On the 3rd day of in vitro culture (DIV3), the non-adherent cells were removed, and fresh complete RPMI 1640 medium was added to the flask. On DIV6 and DIV9, fresh medium was supplemented, and from this day onwards, the medium was changed once a week. The purity of the PMC cultures was verified weekly using toluidine blue staining. After 4 weeks in culture, 0.5 × 10^6^ cells were plated in 24-well plates in growth medium and exposed to vehicle or SP (50 μM) for 24 hours, after which cells were collected and lysed to determine cytokine mRNA expression.

### Quantitative PCR

Full-circumference distal colon (1 cm) was immediately placed in RNAlater solution until RNA isolation. RNA was isolated using the Nucleospin RNA kit (Macherey-Nagel) according to the manufacturer’s protocol. Following isolation, the RNA quality and concentration were determined using the Agilent Bioanalyzer 2100 (Agilent Technologies), and a total of 1μg RNA was reverse transcribed using the iScript cDNA synthesis kit (Bio-Rad Laboratories). RT-qPCR was performed on 2 μL of 1/10 diluted cDNA using the SSO Advanced Universal SYBR Green Supermix (Bio-Rad Laboratories), with a total of 40 amplification cycles and the PCR protocol according to the manufacturer’s instructions. Primer pairs are listed in Table 2. The genes *hprt1* and *rps29* were selected as reference genes, following analysis of their expression stability by GeNorm analysis in the qbase plus 3.0 software (Biogazelle, www.qbaseplus.com). Target gene expression was determined with the qbase plus 3.0 software and represented as normalized expression relative to the control group.

**Table 2:** List of Primers Pairs Used for Quantitative PCR

Target gene	Forward (5′–3′)	Reverse (5′–3′)
*Mrgprb2*	GAGCAAAGGAACATGAGTGGAGA	TTGTTCCTGGTGACACAAACT
*Il1 β*	TCTCGCAGCAGCACATCA	CACACACCAGCAGGTTAT
*Tnf-α*	CCTGTAGCCCACGTCGTAG	GGGAGTAGACAAGGTACAACCC
*Il6*	CCATAGCTACCTGGAGTACATG	TGGAAATTGGGGTAGGAAGGAC
*Cxcl1*	ACTCAAGAATGGTCGCGAGG	GTGCCATCAGAGCAGTCTGT
*Cxcl2*	AGGGCGGTCAAAAAGTTTGC	CGAGGCACATCAGGTACGAT
*Ccl2*	TGCCCTAAGGTCTTCAGCAC	TGCCCTAAGGTCTTCAGCAC
*Ccl3*	GCCACATCGAGGGACTCTTC	GATGGGGGTTGAGGAACGTG
*Ccl4*	CTGTGCAAACCTAACCCCGA	AGGGTCAGAGCCCATTGGT
*Ccl5*	GGAGATGAGCTAGGATAGAGGG	TGCCCATTTTCCCAGGACCG
*Hprt1*	GGTTAAGCAGTACAGCCCCA	GTCTGGCCTGTATCCAACAC
*Rps29*	GCAAATACGGGCTGAACATG	GACTAGCATGATCGGTTCCAC

### Flow Cytometry on Colon Lamina Propria

The colon was isolated from caecum to rectum and was cut into 1-cm pieces after removal of fecal matter. To remove epithelial cells, colon pieces were incubated in HBSS (without Ca^2+^ and Mg^2+^) containing 5 mM EDTA for 40 minutes at 37 °C with continuous shaking. Tissue pieces were then finely minced and digested with 0.5 mg/ml collagenase D and 5 U/ml DNAse in RPMI-1640 supplemented with 2% HEPES and 2% FBS for 30 minutes at 37 °C with continuous shaking. Tissue digests were filtered through a 100 μm strainer and mononuclear cells were further enriched using 40%/80% Percoll gradient centrifugation. Single cell suspensions were blocked with rat anti-mouse CD16/CD32 (Fc block, BD Biosciences) for 10 minutes and afterwards incubated with fluorophore-conjugated anti-mouse antibodies for 20 minutes at 4 °C (Table 1). Dead cells were excluded using Fixable Viability Dye eFluor BV480 (eBiosciences). During flow cytometric acquisition, doublets were excluded. Neutrophils were defined as: CD45+CD11b+Ly6g+. Samples were acquired using a Symphony (BD Biosciences) and analyzed with FlowJo software (version 4.6.2, Treestar).

### Neutrophil Depletion

To perform neutrophil depletion, WT mice were treated with intraperitoneal injections (200 μl) of anti-Ly6G clone 1A8 (1.25mg/ml; Bio X Cell, catalog no. BE0075-1) or isotype antibody (1.25 mg/ml; Bio X Cell, catalog no. BE0089) on days 0, 2, 4, 6, and 8 of the DSS treatment. The resulting effect on neutrophil and macrophage counts was analyzed by flow cytometry on day 10. Samples were prepared and analyzed as explained above. Neutrophils were gated as C45+CD11b+Ly6G+. Macrophages were gated as CD45+CD11b+Ly6g-CD64+Ly6c-MHCII+.

### Myeloperoxidase Activity

MPO activity was measured in colonic tissue as a parameter for neutrophil infiltration.^51^ Briefly, colon (1 cm) was immersed in potassium phosphate buffer (pH 6.0) containing 0.5% hexadecyltrimethylammonium bromide (0.02 mL/mg tissue ratio). Thereafter, samples were homogenized using ceramic beads and subjected to 2 freeze-thawing cycles and subsequently centrifuged at 10,000 g for 15 minutes at 4 °C. Supernatant (0.1 mL) was then added to 2.9 mL of o-dianisidine solution, and the change in absorbance of the samples was read at 460 nm over 60 seconds using a Spectronic Genesys 5 spectrophotometer. MPO activity is defined as the quantity needed to transform 1 μM H_2_O_2_ to H_2_O within 1 minute at 25 °C and is expressed as unit/mg tissue.

### Bulk RNA Sequencing and Bioinformatics

Full-circumference colon was used to isolate RNA using a Nucleospin RNA isolation kit (Macherey-Nagel) according to the manufacturer’s protocol. RNA quality and concentration were measured with an Agilent Bioanalyzer using an RNA nano kit (Agilent Technologies). All samples had RIN values higher than 8. An equivalent of 500 ng RNA was used for cDNA library preparation using a QuantSeq 3′ mRNA-Seq Library Prep kit FWD (Lexogen) and a qPCR add-on kit, all according to the manufacturer’s protocols. Next, cDNA libraries were run on a Fragment Analyzer (Agilent), equimolar pooled, and sequenced using an Illumina NextSeq 500/550 High Output v2.5 kit for 75 cycles. Resulting reads were trimmed using fastp and aligned to the mouse reference genome (mm10) using STAR.^52^ Differentially expressed genes were identified using DESeq2 with standard settings (Benjamini-Hochberg adjusted *P*-value cutoff at 0.1).^53^ Functional annotation, including GeneOntology term enrichment was done using Metascape using default settings.^54^

### Statistical Analysis

Data are represented as mean ± standard error of the mean (SEM). Biological replicates and group sizes are denoted in the figure legends. Differences between groups were statistically analyzed with the unpaired Student *t*-test, 1-way or 2-way analysis of variance (ANOVA), as denoted in the figure legends. Statistical analyses were performed in GraphPad Prism 6 with *P* < .05 significance level. Graphs were made in GraphPad Prism 6.
